# Circulating Tumor Function: A Systems Biology Framework for Liquid Biopsy in Genitourinary Cancers

**DOI:** 10.3390/genes17091035

**Published:** 2026-08-29

**Authors:** Roxana Andra Coman, Andreea Nutu, Lia-Raluca Olari, Stefan Strilciuc, Dana Monica Iancu, Ioana Berindan-Neagoe

**Affiliations:** 1 Department of Fundamental Discipline, Anatomy, Faculty of Nursing and Health Sciences, Iuliu-Hatieganu University of Medicine and Pharmacy, Victor Babes Street Number 8, 400347 Cluj-Napoca, Romania; dr.roxanacoman@yahoo.com (R.A.C.); dr.danamonica@gmail.com (D.M.I.); 2 Department of Urology, MedLife Humanitas Hospital, 400664 Cluj-Napoca, Romania; 3 Genomics Department, MEDFUTURE Institute of Biomedical Research, Iuliu-Hatieganu University of Medicine and Pharmacy, 400336 Cluj-Napoca, Romania; andreeanutu.an@gmail.com (A.N.); raluca.mhm@gmail.com (L.-R.O.); 4 Regional Institute of Gastroenterology and Hepatology “Octavian Fodor”, 19–21 Croitorilor Street, 400162 Cluj-Napoca, Romania; 5 Doctoral School, Iuliu-Hatieganu University of Medicine and Pharmacy, 400012 Cluj-Napoca, Romania; 6 Academy of Medical Sciences, 030171 Bucharest, Romania

**Keywords:** liquid biopsy, circulating tumor function, systems biology, extracellular vesicles, noncoding RNA, redox biology, genitourinary cancer

## Abstract

Liquid biopsy enables minimally invasive detection and longitudinal monitoring of tumor-derived material in blood and urine. In genitourinary cancers, most applications have focused on genomic alterations in circulating tumor DNA (ctDNA), together with circulating tumor cells (CTCs), extracellular vesicles (EVs), and cell-free RNAs. These measurements are clinically informative but are often interpreted as isolated, predominantly descriptive biomarkers and therefore incompletely represent the adaptive processes that determine progression and treatment response. We propose circulating tumor function (CTF) as a systems biology framework for integrating tumor-derived and host-derived genomic, regulatory, metabolic, redox, and immune signals obtained through serial liquid biopsy. CTF is not a single analyte or assay; rather, it is an inference model intended to generate interpretable functional states, including proliferative activity, immune evasion, metastatic potential, metabolic stress, and therapeutic adaptation. We review the contributions and limitations of ctDNA, ncRNA networks, EV-mediated signaling, redox biomarkers, and tumor–host crosstalk in prostate, bladder, renal, and testicular cancers. We also outline the analytical and clinical validation required to determine whether integrated CTF models provide incremental value over established single-analyte approaches. This framework may help reposition liquid biopsy from molecular detection toward functional precision oncology.

## 1. Introduction: From Detection to Interpretation

Cancer remains a major global cause of morbidity and mortality [1]. Although tissue biopsy is the diagnostic reference standard for many malignancies, it is invasive, may be difficult to repeat, and can be limited by sampling bias and spatial tumor heterogeneity. These constraints reduce its suitability for early detection and longitudinal assessment of evolving disease [2].

Liquid biopsy (LB) refers to the analysis of tumor-associated cells, nucleic acids, vesicles, proteins, metabolites, or other molecular features in blood, urine, and additional body fluids [3]. Blood and urine are particularly suitable for repeated sampling, creating opportunities for minimally invasive screening, molecular characterization, treatment monitoring, and detection of residual or recurrent disease [4,5].

LB has attracted particular interest in genitourinary oncology because prostate, bladder, renal, and testicular cancers collectively represent a substantial proportion of solid tumors and can be sampled through both blood and urine [6]. Prostate cancer (PCa) is one of the most frequently diagnosed malignancies in men [7]. Prostate-specific antigen (PSA) testing, multiparametric magnetic resonance imaging, and targeted or systematic biopsy remain central to its diagnostic pathway, but limited specificity, overdiagnosis, sampling error, and biopsy-related morbidity continue to motivate complementary biomarkers [8].

Bladder cancer (BC) is characterized by frequent recurrence and prolonged surveillance; cystoscopy remains the reference procedure but is invasive and poorly tolerated, contributing to substantial clinical and economic burden [9]. Renal cell carcinoma (RCC) accounts for most kidney malignancies and presents additional challenges because biopsy may be limited by anatomical access, intratumoral heterogeneity, and procedural risk, while imaging does not directly capture evolving molecular states or minimal residual disease [10,11].

Testicular germ cell tumors (TGCTs) are uncommon overall but are the most frequent solid malignancies in adolescent and young adult men, and their incidence has increased in several populations [12]. Outcomes are excellent for localized disease and remain favorable in many metastatic cases; nevertheless, relapse and treatment resistance occur in a clinically important minority, making sensitive post-treatment surveillance essential [13].

Across these malignancies, LB provides a minimally invasive window into tumor biology through analytes that include cell-free DNA (cfDNA), circulating tumor DNA (ctDNA), circulating tumor cells (CTCs), cell-free RNAs, and extracellular vesicles (EVs) [14,15]. The accessibility of blood and urine supports serial sampling and potentially more timely assessment of disease evolution than episodic tissue biopsy alone [16]. Clinical maturity, however, varies substantially by cancer type, disease stage, analyte, and intended use.

Most current LB strategies prioritize the detection or quantification of tumor-associated alterations, especially ctDNA mutations and methylation patterns. These readouts can reveal tumor burden, clonal evolution, and resistance-associated genomic changes, but they do not by themselves capture the full range of adaptive processes that influence therapy response, immune evasion, metastatic progression, and metabolic plasticity [17]. This limitation is particularly relevant in genitourinary oncology, where distinguishing indolent from aggressive disease and predicting treatment response remain persistent clinical challenges [6].

The next phase of LB development therefore requires not only more sensitive detection, but also a framework for biological interpretation. Rather than asking only which tumor-associated features are present, functional liquid biopsy should address what the tumor and host are doing over time. In this concept-driven narrative review, we introduce circulating tumor function (CTF) and examine how genomic and epigenomic alterations, noncoding RNA (ncRNA) networks, EVs, redox biology, metabolism, and immune interactions could be integrated into longitudinal models of tumor state. We then assess the translational readiness of these components in PCa, BC, RCC, and TGCTs.

This narrative review was conducted through systematic searches of PubMed/MEDLINE, with supplementary searches of Scopus and Google Scholar. The primary search used the following MeSH terms and free-text keywords individually and in combination: ‘liquid biopsy’, ‘circulating tumour DNA’, ‘cell-free DNA’, ‘circulating tumour cells’, ‘extracellular vesicles’, ‘exosomes’, ‘non-coding RNA’, ‘microRNA’, ‘long non-coding RNA’, ‘redox’, ‘reactive oxygen species’, ‘systems biology’, ‘multi-omics’, ‘genitourinary cancer’, ‘prostate cancer’, ‘bladder cancer’, ‘renal cell carcinoma’ and ‘testicular germ cell tumour’. The reference lists of the identified articles were manually screened for any further relevant publications. The selection of references reflects the authors’ expert judgement and is subject to the inherent limitations of narrative review methodology, including potential selection bias.

## 2. Limitations of Current Liquid-Biopsy Paradigms

The most intensively studied LB analytes include ctDNA, CTCs, cell-free RNAs, and EVs. Circulating proteins and metabolites provide additional information, but they are less consistently integrated with tumor-specific molecular readouts. Each analyte captures a different aspect of disease biology and has distinct pre-analytical, analytical, and interpretive limitations.

ctDNA is the tumor-derived fraction of cfDNA and can be detected in plasma and, depending on tumor location, in urine and other body fluids [18]. Mutation, copy-number, fragmentomic, and methylation analyses can inform diagnosis, clonal evolution, and resistance. Sensitivity is nevertheless constrained by low tumor shedding, particularly in early-stage or low-volume disease, and genomic composition alone is an incomplete proxy for the current functional state of a tumor [19].

CTCs are intact cancer cells released from primary or metastatic lesions into the circulation [20]. They can provide cellular, morphological, genomic, transcriptomic, and phenotypic information, but their rarity, short persistence in peripheral blood, biological heterogeneity, and platform-dependent recovery limit routine application [21].

Cell-free RNAs provide regulatory information that is not available from DNA alone. MicroRNAs (miRNAs) are short ncRNAs, typically 18–23 nucleotides in length, that regulate transcript stability and translation [22]. Long noncoding RNAs (lncRNAs) are transcripts longer than 200 nucleotides with diverse transcriptional, post-transcriptional, and epigenetic functions [23]. Circular RNAs and messenger RNAs add further regulatory and expression-level information, although abundance, normalization, and tissue-of-origin attribution remain challenging.

EVs are lipid-bilayer particles released by most cell types and can transport DNA, RNA, proteins, lipids, and metabolites. Their relative stability and multimolecular cargo make them attractive sources of integrated biological information. However, EV populations are heterogeneous; tumor-derived vesicles may represent only a small fraction of total circulating EVs, and isolation and characterization methods remain insufficiently standardized [24].

These analytes therefore provide complementary but incomplete views of tumor biology. When analyzed in isolation, they rarely reconstruct the interacting molecular processes that underlie tumor behavior. The central challenge is to integrate orthogonal signals without losing analytical rigor, biological interpretability, or clinical feasibility.

## 3. Defining Circulating Tumor Function

We define circulating tumor function (CTF) as an inferred, time-dependent representation of biologically active tumor and tumor–host states reconstructed from circulating molecular and cellular signals. CTF extends beyond the detection of static genomic alterations by integrating tumor-derived and host-derived information from ctDNA, ncRNAs, EVs, circulating proteins and metabolites, redox markers, immune mediators, and, where feasible, CTC phenotypes. The intended outputs are interpretable functional states relevant to proliferation, immune modulation, metastatic competence, metabolic adaptation, treatment response, and resistance.

**Figure 1:**
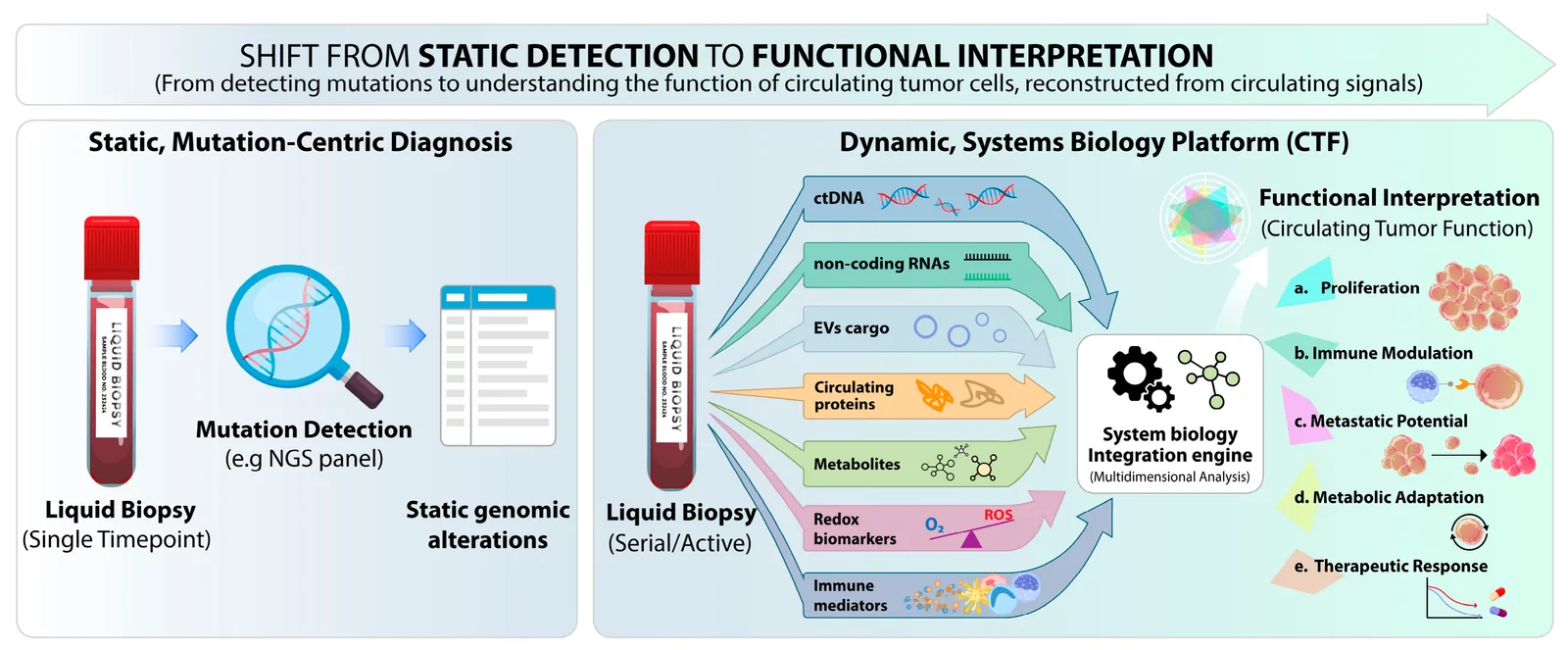
Evolution of liquid biopsy from mutation detection to functional interpretation. Conventional liquid biopsy frequently centers on the detection of tumor-associated genomic alterations. The proposed CTF framework integrates longitudinal ctDNA, ncRNA, EV, protein, metabolite, redox, and immune measurements to infer dynamic tumor and tumor–host states, including proliferation, immune modulation, metastatic potential, metabolic adaptation, and therapeutic response. Abbreviations: CTF, circulating tumor function; ctDNA, circulating tumor DNA; EV, extracellular vesicle; ncRNA, noncoding RNA; NGS, next-generation sequencing.

CTF is not synonymous with a multi-analyte panel. A CTF model should link measurements across biological layers, incorporate serial sampling, and produce a functional interpretation that can be tested against clinical outcomes. Figure 1 illustrates this transition from mutation-centered detection to dynamic systems-level inference. The framework is deliberately hypothesis-generating: whether it improves accuracy, timeliness, or clinical utility over optimized single-analyte LB must be established empirically.

A minimum CTF model must satisfy three criteria to differ from a conventional longitudinal multi-analyte panel. Tumor-Anchored Signal Hierarchy: the model must include at least one tumor-specific anchor—a somatic ctDNA alteration, tumour-enriched methylation pattern or tumour-derived RNA/EV feature—serving as the reference against which all other layers are interpreted. Without this anchor, the model cannot reliably distinguish tumour-driven variation from nonspecific host response. Functional State Inference via a Predefined Network Model: scores—such as proliferative activity index or immune evasion score—must be derived from mechanistically informed priors and known regulatory relationships. Serial Measurement Linked to a Predefined Clinical Decision: two time-anchored measurements must be linked to a specific, prospectively defined clinical decision point. A single multi-omic snapshot does not constitute CTF. This requirement aligns with the longitudinal ctDNA dynamics paradigm and is what operationally distinguishes CTF from cross-sectional profiling.

Quantitative inference of functional states uses a three-layer computational architecture. In the first layer, signals are normalized against their pre-treatment baseline and technical controls. This addresses inter-individual biological variation and pre-analytical noise. In the second layer, signals are weighted by the ctDNA tumor fraction or a tumor-anchor metric. A rise in markers of systemic oxidative stress with a stable or declining ctDNA tumor fraction would receive a low tumor-specificity weight, flagged as potentially nonspecific, directly addressing the challenge of distinguishing tumor-derived from host-derived signals. In the third layer, signals are projected onto a predefined biological network, allowing incorporation of new measurements while preserving temporal trajectory information.

One of the main challenges in using CTF is distinguishing signals from the tumour itself from signals from the rest of the body. Many different cell types produce signals that can be measured in the blood, and these are influenced by factors like inflammation, infection, kidney function, medications, diet, age, and existing health problems. To work out the origin of a tumour, we can look at three things: (i) how a signal changes over time in relation to the tumour; (ii) the proteins on the outside of the EVs; and (iii) comparing signals with the tissue and urine to see if they match. These strategies do not completely solve the problem, but they give us a clear approach to dealing with it.

To be validated, any CTF model must demonstrate analytical performance, concordance with tumour biology, reproducibility across sites and platforms, and incremental clinical utility over standard biomarkers and clinical models.

Table 1 contrasts conventional, predominantly analyte-centered LB with the proposed CTF framework. The distinction is conceptual rather than absolute: existing LB assays can contribute to CTF when they are integrated longitudinally and interpreted in relation to biological function.

### 3.1. Redox Biology as an Underused Functional Layer

Redox homeostasis is the dynamic balance between the production and elimination of reactive oxygen species (ROS). It influences signal transduction, genomic stability, proliferation, cell death, metastatic behavior, and treatment response. Despite this mechanistic relevance, redox-associated measurements are rarely incorporated into LB models [25]. Redox biomarkers are less tumour-specific, standardised and validated than ctDNA and established urinary molecular assays and so should be used to explore the CTF rather than as standalone tumour-specific biomarkers or alternatives to liquid-biopsy assays.

ROS and antioxidant systems, including the glutathione axis (GSH/GSSG), can influence transcriptional and post-transcriptional regulation. Candidate circulating readouts include oxidative DNA-damage products, lipid-peroxidation metabolites, thiol-redox measures, and antioxidant enzyme activities [26]. These markers may report systemic oxidative stress and metabolic adaptation, but they are not inherently tumor-specific and can be affected by inflammation, comorbidities, diet, medication, specimen handling, and storage. Their use in CTF will therefore require strict pre-analytical control and anchoring to tumor-derived signals.

**Figure 2:**
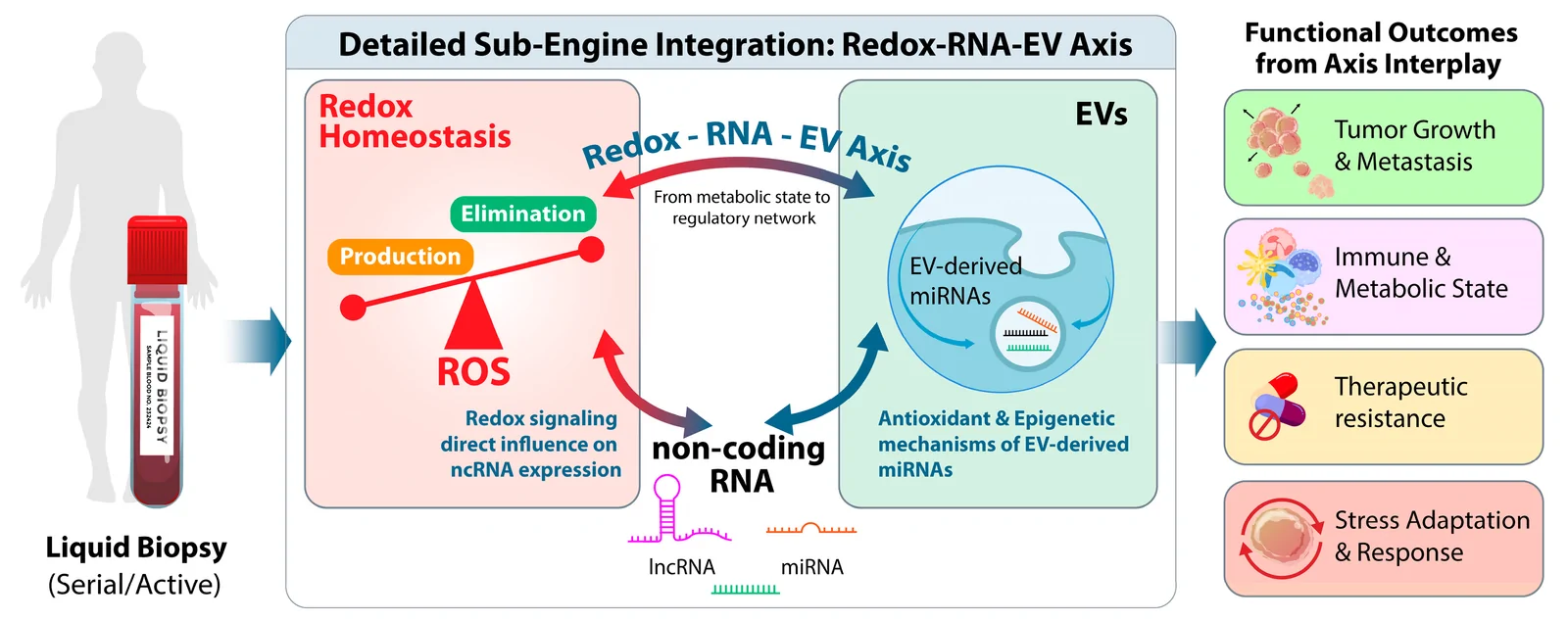
The redox–RNA–extracellular-vesicle axis in CTF. Redox homeostasis, ncRNA regulation, and EV-mediated transfer form an interconnected module through which metabolic and epigenetic signals may influence four broad functional outcome categories: tumor growth and metastasis, immune and metabolic state, therapeutic resistance, and stress adaptation and response. Abbreviations: CTF, circulating tumor function; EV, extracellular vesicle; lncRNA, long noncoding RNA; miRNA, microRNA; ncRNA, noncoding RNA; ROS, reactive oxygen species.

ROS, miRNAs, and EVs interact within the tumor microenvironment to influence angiogenesis, immune responses, and therapeutic resistance [27,28] (Figure 2). EV-associated miRNAs may modify oxidative stress through antioxidant and epigenetic pathways, although heterogeneous EV methods and incomplete mechanistic annotation continue to impede clinical translation [29]. Redox signaling can also alter ncRNA expression, linking metabolic state to regulatory networks. For example, an eight-lncRNA redox-related signature was reported to stratify outcomes in BC [30]. Such findings support redox biology as a candidate functional layer, but circulating validation and demonstration of incremental clinical value remain necessary.

### 3.2. Noncoding RNA Networks as Functional Mediators

ncRNAs, including miRNAs, lncRNAs, and circular RNAs, regulate gene expression through post-transcriptional, transcriptional, and epigenetic mechanisms. They operate within networks rather than as isolated biomarkers and can coordinate proliferation, apoptosis, epithelial-to-mesenchymal transition, immune regulation, metabolic reprogramming, and therapeutic resistance. These functions make ncRNAs plausible reporters and mediators of tumor plasticity [31,32].

Circulating ncRNAs originate from tumor, stromal, immune, endothelial, and other host cells. Encapsulation within EVs or association with RNA-binding proteins can protect them from degradation and enhance their stability in biological fluids [31,33]. This stability supports biomarker development, but cellular origin, hemolysis, normalization, and platform effects must be considered when interpreting circulating signatures.

Within CTF, ncRNA measurements would be interpreted in relation to their regulatory targets and to other circulating layers rather than used solely as classification features. Integration with ctDNA, EV cargo, proteomic, metabolomic, redox, and immune data may enable longitudinal assessment of progression, response, and resistance [34]. Mechanistic network priors may also improve interpretability and reduce the risk that high-dimensional signatures function as opaque statistical classifiers.

### 3.3. Extracellular Vesicles as Carriers of Tumor Function

EVs are nano- to microscale lipid-bilayer particles secreted by nearly all cell types and increasingly recognized as active mediators of intercellular communication. Their cargo can include DNA, multiple RNA classes, proteins, lipids, glycans, and metabolites, providing a multimolecular representation of the cell of origin and its physiological state [35].

In genitourinary malignancies, EVs have been implicated in tumor initiation, progression, immune modulation, angiogenesis, metastatic dissemination, and drug resistance. Their stability and biocompatibility have also stimulated interest in diagnostic, monitoring, and therapeutic applications in PCa, BC, RCC, and TGCTs [36].

Urinary EVs are particularly attractive because they can be sampled noninvasively and may be enriched for signals from the genitourinary tract. RNA, protein, and glycan features in urinary EVs have shown diagnostic or monitoring potential in prostate, bladder, and renal cancers. In the CTF framework, EVs are treated as signaling units that may connect genomic, transcriptomic, proteomic, metabolic, and immune information [37]. Reliable tumor-of-origin assignment and standardized isolation remain prerequisites for clinical implementation.

### 3.4. Tumor–Immune–Circulatory Crosstalk

Tumors communicate bidirectionally with immune, stromal, endothelial, and hematologic compartments through the circulation [38]. Tumor-derived EVs, ctDNA, ncRNAs, and soluble factors coexist with immune cells, platelets, cytokines, chemokines, complement proteins, adhesion molecules, and coagulation factors, forming a systemic signaling network [38,39]. This network can influence immune surveillance, inflammation, vascular remodeling, metastatic dissemination, and treatment adaptation [40]. Circulating EVs may reprogram immune phenotypes, promote endothelial dysfunction, support pre-metastatic niches, and contribute to checkpoint regulation and immune evasion [39,41]. These effects are further shaped by systemic metabolic and redox states [38,42].

A functional circulating model must therefore integrate tumor-derived signals with host responses rather than treating each class as an independent biomarker [38,39]. Longitudinal combinations of tumor burden, immune activation or suppression, endothelial injury, inflammation, coagulation, and metabolic stress may provide a more complete view of progression, immunotherapy response, metastatic risk, and emerging resistance [41,42]. The major interpretive challenge is to distinguish tumor-driven changes from nonspecific systemic variation.

### 3.5. Systems Biology and Multi-Omic Integration

The multidimensional nature of circulating tumor and host signals requires analytical methods that can integrate heterogeneous data while preserving temporal information and biological interpretability.

Multi-omic approaches combine genomic, epigenomic, transcriptomic, proteomic, metabolomic, and immune-derived data to identify interactions that cannot be resolved through single-modality analyses. In precision oncology, such approaches have refined molecular classification, supported biomarker and target discovery, and improved patient stratification [43]. In genitourinary LB, however, the evidence remains fragmented across cohorts, platforms, analytes, and clinical contexts, and methodological heterogeneity limits direct comparison and reproducibility [44].

Machine learning (ML) and network-based models can capture nonlinear associations among molecular layers and may predict progression, response, or resistance [45]. Graph-based methods can incorporate known molecular interactions and thereby improve mechanistic interpretation [43]. Nevertheless, small cohorts, high dimensionality, batch effects, missing data, class imbalance, and overfitting can produce unstable signatures. Model development should therefore include predefined clinical questions, transparent feature selection, nested validation, external testing, calibration, and comparison with established clinical and single-analyte benchmarks.

This vision is related to the digital-twin concept, in which serial molecular, imaging, and clinical data update a patient-specific model of tumor behavior [46]. For CTF, a practical near-term objective is more modest: validated longitudinal functional scores that are analytically reproducible, biologically interpretable, and actionable at defined clinical decision points. Full digital twins will require substantially greater data harmonization, prospective validation, computational infrastructure, and governance.

## 4. Clinical Implications: Toward Functional Precision Oncology

Functional LB could extend precision oncology beyond static genotyping by characterizing biologically active tumor and host states over time. Potential applications include earlier detection, discrimination of indolent and aggressive disease, treatment selection, assessment of minimal residual disease, and early identification of therapeutic adaptation. These benefits remain conditional on proof that integrated models add clinically meaningful information beyond standard care and optimized single-analyte assays. The following sections evaluate the available building blocks and remaining gaps in four genitourinary malignancies.

### 4.1. Liquid Biopsy in Prostate Cancer

The diagnosis, prognosis, and monitoring of PCa have expanded beyond serum PSA, with blood- and urine-based assays providing access to tumor-derived nucleic acids and vesicles. Circulating and EV-associated miRNAs, including miR-21, miR-375, and miR-182-5p, have shown associations with androgen-receptor signaling, microenvironmental remodeling, and disease aggressiveness [47,48]. Urinary RNA assays targeting *PCA3* and *TMPRSS2:ERG* have demonstrated clinical utility in selected diagnostic settings [49]. The urine exosome-based ExoDx Prostate IntelliScore (EPI) has shown high sensitivity and negative predictive value in biopsy-naïve populations and may reduce unnecessary biopsies [50,51,52]. ML supported multimarker approaches, such as PROSTest, are also being investigated to improve specificity after an elevated PSA result [53]. Regarding imaging, integrating miRNAs (let-7a-5p and miR-103a-3p), and PSA with MRI did not provide additional diagnostic value over MRI alone for identifying PCa and clinically significant PCa [54].

ctDNA is difficult to detect in many patients with localized PCa, but cfDNA methylation, fragmentomic, and low-pass whole-genome approaches may improve sensitivity [55]. Urinary RNA technologies are also advancing; the AVATAR platform was developed as a rapid, decentralized biosensor for molecular profiling of urinary PCa biomarkers [56]. These approaches require prospective comparison with contemporary imaging and biopsy pathways.

CTCs have limited sensitivity for early-stage PCa and biochemical recurrence, whereas sensitive tumor-informed ctDNA assays may help identify aggressive disease or increased recurrence risk [57]. In advanced PCa, ctDNA is the most mature LB analyte for detecting actionable alterations and monitoring molecular evolution. CTCs and EVs provide complementary cellular and regulatory information, while methylation and fragmentomic approaches may improve detection but remain vulnerable to low tumor fraction and clonal hematopoiesis [58]. Baseline CTC counts are prognostic in metastatic hormone-sensitive disease [59]. In castration-resistant PCa, higher ctDNA tumor fraction and genomic instability are associated with poorer outcomes, and ctDNA can identify actionable DNA-repair alterations, including *BRCA1* and *BRCA2* changes [60,61].

Serial CTC and ctDNA measurements can add temporal information. Declining CTC counts after treatment are associated with improved survival, whereas persistently elevated counts indicate adverse prognosis [62,63]. Rising ctDNA burden may precede radiographic progression, and detectable ctDNA after local therapy is associated with recurrence risk [60,64]. Molecular imaging and artificial-intelligence methods may further refine diagnosis, risk stratification, and treatment guidance, but their integration with LB requires prospective validation [65].

Some LB assays have entered clinical practice. FoundationOne Liquid CDx supports broad genomic profiling and can identify homologous-recombination-repair alterations relevant to PARP-inhibitor therapy in metastatic castration-resistant PCa [66]. Plasma cfDNA can identify actionable DNA-repair defects in a subset of advanced cases [61,67]. In localized disease, urine-based assays such as EPI can support biopsy decision-making [51,52]. These applications demonstrate the value of specific analytes for defined indications rather than a fully integrated CTF model [59,68].

Redox imbalance is implicated in PCa progression, genomic instability, and treatment resistance, whereas physiological ROS levels participate in normal signaling. Interactions between ROS and miRNA regulation provide a mechanistic rationale for including redox status in functional models [69]. At present, however, redox biomarkers are not sufficiently standardized or tumor-specific for routine PCa LB.

Multi-omic integration has been used to define stemness-related PCa subtypes with different prognoses and predicted responses to androgen-deprivation therapy, taxanes, and immune-checkpoint inhibition [44,70]. Additional analyses have implicated sensory-perception pathways and TNF–CCL20 signaling in therapeutic resistance and chemosensitivity [71], while reviews of metastatic castration-resistant PCa have summarized genomic, transcriptomic, and proteomic biomarkers relevant to precision treatment [72]. Most of these studies are tissue-centered and remain to be translated into serial circulating models.

PCa therefore provides many measurable components for CTF, including genomic alterations, CTC phenotypes, urinary RNAs, and EV-associated signals. Their clinical maturity is uneven: selected genomic and urine assays are used for defined decisions, whereas most ncRNA, EV, redox, and multi-omic models remain investigational. The primary opportunity is not simply to detect more analytes, but to test whether their longitudinal integration improves risk classification and treatment adaptation.

### 4.2. Liquid Biopsy in Bladder Cancer

BC is particularly suited to urine-based LB because surveillance is frequent and cystoscopy is invasive. Urine cytology is highly specific but has limited sensitivity for low-grade disease. Tumor-derived DNA and RNA assays generally provide higher sensitivity and negative predictive value and may support less invasive surveillance in selected patients [73,74,75,76]. Molecular urine assays are most valuable when used alongside conventional cytology. A multicentre study showed that using Bladder EpiCheck^®^ and cytology together is the best way to follow up with patients with high-grade non-muscle-invasive BC. This approach reduces the number of unnecessary invasive procedures needed [77].

Among extensively studied tests, Bladder EpiCheck^®^, a DNA-methylation panel, and Xpert^®^ Bladder Cancer Monitor, an mRNA assay, have generally outperformed cytology and UroVysion in sensitivity, particularly for high-grade disease [73,74,78]. Cxbladder^®^ assays have shown utility in diagnostic, triage, and follow-up settings [79,80]. Urinary cfDNA assays targeting alterations such as *TERT*, *FGFR3*, *PIK3CA*, and *ERBB2* and the lower-cost Uromonitor^®^ assay have also shown promising performance [81,82]. Urine DNA-methylation approaches may detect early-stage, recurrent, or residual disease [83]. Photothermal infrared imaging has been proposed as an alternative method for detecting tumor cells in urine [84]. In addition, urinary-cell transcripts such as *MDK* and *KRT17* have been associated with non-muscle-invasive BC detection [85]. Urinary H4C6 and TWIST1 gene methylation, alongside VI-RADS scoring, demonstrate high diagnostic accuracy in predicting residual tumours following transurethral resection of the bladder in patients with non-muscle-invasive BC [86].

EV- and exosome-based biomarkers provide complementary information [87]. EV-associated RNAs and exosomal miRNA panels have shown associations with recurrence and may be useful when DNA-based results are equivocal [88,89]. Artificial intelligence (AI) supported EV pipelines have also been proposed for molecular subtyping and prognosis [90], but these studies require independent validation and harmonized EV workflows.

Plasma and urinary ctDNA extend LB beyond surveillance by enabling assessment of residual disease, relapse risk, and treatment response [91,92]. The utLIFE approach combines shallow whole-genome sequencing, targeted sequencing of 155 genes, and ML classification; it showed diagnostic and prognostic potential for residual disease and recurrence in non-muscle-invasive BC [93].

ctDNA clearance during neoadjuvant therapy has been associated with improved outcomes. Exploratory analyses from systemic and adjuvant immunotherapy studies, including KEYNOTE-361 and IMvigor010, suggest that ctDNA dynamics may enrich prognostic assessment and identify groups more likely to benefit from treatment [94,95]. Post-cystectomy ctDNA is prognostic and may detect recurrence before conventional assessment [83,96,97]. In metastatic BC, circulating analyses can also extend beyond DNA alterations to clinically relevant expression programs [98]. Prospective trials such as IMvigor011, TOMBOLA, and MODERN are evaluating ctDNA-guided management strategies [99].

Compared with UroVysion fluorescence in situ hybridization, several newer urine assays provide higher negative predictive values and improved sensitivity, supporting their potential as rule-out tools that could reduce cystoscopy frequency in carefully selected settings [76]. Overall, urine DNA, RNA, and EV assays together with plasma ctDNA are expanding diagnostic, prognostic, treatment-selection, and surveillance options [100]. Their substitution for cystoscopy or cytology, however, requires indication-specific prospective evidence.

Oxidative stress contributes to BC biology, and altered ROS production and antioxidant defenses have been reported in tumor tissue [101,102]. Redox pathways are being investigated as therapeutic targets, but tissue findings cannot be assumed to translate directly into tumor-specific circulating markers. Serial redox measurements would need to be interpreted alongside tumor-derived urinary or plasma signals.

Multi-omic studies have identified prognostic markers, molecular subtypes, and therapeutic vulnerabilities in BC. The consensus molecular classification defines six biologically and clinically relevant subtypes [103]. Integrative analyses have proposed biomarkers such as *GARS1* and additional prognostic signatures associated with survival or treatment response [104,105]. Sex-associated molecular and immune features also suggest a potential role for sex-informed biomarker development and treatment stratification [106]. Translation into LB will require circulating surrogates of these tissue-defined states.

Among the four cancers considered, BC has the most mature urine-based LB ecosystem, complemented by plasma ctDNA for residual-disease and treatment-response assessment. Nevertheless, maturity is concentrated in nucleic-acid assays. EV, ncRNA, redox, immune, and integrated multi-omic readouts remain less standardized. BC is therefore a strong setting in which to test whether CTF adds value to already effective single-analyte and multimarker assays.

### 4.3. Liquid Biopsy in Renal Cell Carcinoma

RCC presents major biomarker challenges because of intratumoral heterogeneity, stromal complexity, difficult access to some lesions, and variable tissue sampling [107,108,109]. Plasma ctDNA yields are lower than in many other advanced solid tumors, with reported detection rates varying by stage, burden, assay, and biological subtype [110,111]. Tumor-informed and ultra-deep sequencing approaches may improve sensitivity in higher-stage disease [112]. Translocation RCC, which is driven by diverse *TFE3* fusions, can be difficult to identify; cfDNA epigenomic profiling may support noninvasive detection and monitoring [113,114].

Cell-free and EV-associated RNAs may complement ctDNA in RCC, particularly in low-shedding disease. miR-210 is among the most frequently evaluated candidates in urine and plasma, with reported diagnostic sensitivity and specificity in the approximately 75–83% range [115]. Additional lncRNA and miRNA signatures have shown prognostic or diagnostic potential [116]. Urinary miRNAs, including miR-15a, miR-15b, miR-16, miR-210, and let-7b, have also been investigated for clear-cell RCC diagnosis and postoperative monitoring [117]. These findings remain heterogeneous and require standardized normalization and independent validation.

Early studies of clear cell RCC demonstrated associations between computed tomography radiomic features and miRNA expression, particularly of miR-21-5p and miR-15a [118,119]. Machine learning-based radiogenomic approaches could facilitate non-invasive molecular tumour characterisation, thereby supporting improved stratification and personalised RCC management.

Molecular barcoding, ultra-deep sequencing, fragmentomics, and methylation profiling are improving ctDNA detection in low-shedding RCC [120,121]. Serial tumor-informed ctDNA has shown potential for monitoring response and identifying progression in patients receiving systemic therapy, including immunotherapy [122,123].

Urinary EVs are informative analytes across renal diseases and may provide a biologically plausible route to RCC-derived RNA and protein signals [124]. A meta-analysis supports diagnostic potential for exosomal ncRNAs in RCC [125], although study heterogeneity, small cohorts, and nonuniform EV methods limit immediate clinical translation.

LB may also support treatment monitoring and detection of residual disease. Across solid tumors, changes in ctDNA during therapy are associated with outcome, but the magnitude and reliability of this association are cancer- and assay-dependent [126,127]. In RCC, longitudinal ctDNA studies and ultrasensitive assays have linked molecular response or postoperative positivity with progression and recurrence risk [122,123,128]. Tumor-informed strategies generally appear more informative than tumor-agnostic approaches, although sensitivity remains limited. Circulating kidney-injury molecule-1 has also shown potential in clear-cell and papillary RCC settings [129].

ROS have context-dependent roles in RCC. Physiological ROS participate in signaling, whereas persistent redox imbalance can promote oxidative damage, metabolic reprogramming, immune alteration, and progression. Oxidative-stress-related molecular signatures have shown prognostic associations in clear-cell RCC [130], and metabolic reprogramming is central to RCC pathogenesis and therapeutic vulnerability [131]. These observations are consistent with broader links between redox homeostasis, tumor progression, immunity, and treatment response [132]. The key translational challenge is to derive stable, tumor-relevant circulating redox readouts.

Integrative genomic, transcriptomic, and immune analyses have identified programmed-cell-death patterns in clear-cell RCC with distinct prognoses and immune landscapes. Anoikis-associated high-risk tumors showed genomic instability and immunosuppression, whereas a lysosome-dependent-cell-death pattern was associated with more favorable antitumor immunity; *HMOX1* and *PIK3CG* were proposed as candidate targets [133]. Proteogenomic analyses have also linked BAP1-deficient tumors to increased c-MET expression and potential sensitivity to c-MET inhibition [134]. These findings illustrate functional states that could eventually be monitored through circulating surrogates.

RCC is a demanding test case for CTF because the genomic anchor that supports LB in other cancers is weakened by low and variable ctDNA shedding. ncRNAs, EVs, proteins, metabolites, and host-response measures may therefore contribute proportionally more information. This opportunity also increases the risk of nonspecific signals, making tumor-of-origin attribution and multimodal validation especially important.

### 4.4. Liquid Biopsy in Testicular Germ Cell Tumors

ctDNA is being investigated in TGCTs, particularly for patients without elevated conventional serum markers such as alpha-fetoprotein or human chorionic gonadotropin. Early studies of actin-β DNA fragments, mitochondrial DNA fragmentation, and methylation panels reported diagnostic discrimination across seminoma and nonseminoma subtypes, while KIT ligand CpG methylation has been proposed as a seminoma-associated marker [135]. These approaches remain exploratory.

Highly sensitive residual-disease assays may detect tumor-specific alterations after orchiectomy or during surveillance [111]. ctDNA levels have been associated with stage and disease activity and may complement conventional serum markers [136]. Post-treatment ctDNA positivity has also been linked to relapse risk, but ctDNA is not yet as clinically developed as miR-371a-3p for TGCT diagnosis and monitoring [137]. Candidate biomarkers including *CLDN6*, cfDNA/ctDNA features, and the miR-371–373 cluster are expanding the range of noninvasive approaches [138].

CTCs have limited diagnostic utility in TGCTs because early detection rates are low, enrichment is technically difficult, and tumor-specific markers are not standardized [139]. Higher CTC counts have been associated with advanced stage and adverse outcomes, but the evidence base remains small [140].

miRNAs are the most promising circulating biomarkers in TGCTs. miR-371a-3p has shown high diagnostic accuracy in seminoma and nonseminoma, with reported sensitivity of approximately 90–96% and specificity of 84–100%, but it does not reliably detect teratoma [137,141]. Levels correlate with tumor burden and decline rapidly after orchiectomy, supporting potential roles in diagnosis, staging, treatment monitoring, and relapse detection. Research remains dominated by this single marker, with limited AI-supported or multi-omic integration [44]. Circulating miRNA testing shows high patient acceptability and potential utility for diagnosis, monitoring, and prognostication of malignant testicular GCTs, potentially reducing reliance on serial CT imaging, although practical barriers to clinical implementation remain [142,143]. Broader validation and assay standardization are still needed before routine implementation [144].

Systemic inflammation and oxidative stress may also contribute to TGCT progression. Preoperative inflammatory and redox biomarkers have been associated with tumor stage and metastatic potential and could potentially complement established prognostic measures [145]. Their specificity and added value relative to miR-371a-3p and conventional markers remain unknown.

TGCTs currently provide the narrowest foundation for CTF among the four malignancies reviewed. The strong performance of miR-371a-3p has reduced the immediate incentive to develop broader panels, while EV-based, immune, redox, and multi-omic approaches remain underexplored and CTC analysis has limited clinical utility. A rational CTF strategy would therefore test whether complementary host-response or functional signals improve detection of teratoma, residual disease, relapse, or treatment resistance beyond miR-371a-3p alone.

Table 2 summarizes representative molecular and multi-omic strategies relevant to CTF in genitourinary cancers. Most were developed from tissue-based or single-analyte datasets and should be viewed as candidate building blocks rather than validated circulating functional models.

## 5. Limitations and Translational Requirements

Individual LB analytes have different sensitivity, specificity, and technical constraints. CTCs are rare and phenotypically heterogeneous, making enrichment and characterization difficult and costly [147]. ctDNA concentrations are often low in early-stage disease, and collection, processing, storage, sequencing depth, clonal hematopoiesis, and background cfDNA can affect results [148]. EV isolation is complicated by heterogeneity in size, cellular origin, and cargo, while circulating RNAs are sensitive to pre-analytical variation and require robust normalization. Because normal and tumor cells both release EVs and ncRNAs, tumor specificity can be limited [24,149].

Integration introduces additional challenges. Multi-omic datasets often combine platforms with different dynamic ranges, batch structures, missingness patterns, and sampling requirements. High dimensionality relative to cohort size increases overfitting risk, while black-box models may generate apparently accurate predictions without providing stable biological interpretation. CTF development should therefore use prespecified endpoints, harmonized collection protocols, locked analytical pipelines, external validation, and direct comparison with simpler models [16,33,43,44,45,46].

Clinical implementation will also depend on feasibility, turnaround time, cost, equity of access, regulatory qualification, and evidence that acting on a CTF result improves patient outcomes. Tumor-of-origin attribution is particularly important because inflammation, renal function, age, medication, and other host factors can influence circulating immune, metabolic, redox, RNA, and EV signals. Prospective studies should define sampling intervals and decision thresholds in advance and should evaluate whether integrated models provide meaningful net benefit rather than only higher statistical discrimination.

## 6. Conclusions

Liquid biopsy is becoming an important component of precision oncology, but progress from detection to functional interpretation requires more than the simultaneous measurement of additional biomarkers. CTF is proposed here as a systems biology framework in which longitudinal tumor-derived and host-derived signals are integrated to infer interpretable biological states. Across genitourinary cancers, genomic readouts are most mature in advanced PCa and BC, urine-based nucleic-acid assays have particular relevance in PCa and BC, ctDNA sensitivity remains a major limitation in RCC, and miR-371a-3p dominates the TGCT landscape. EV, ncRNA, immune, metabolic, and redox measurements provide plausible complementary layers, but most are not yet standardized or clinically validated. The central research question is therefore whether a predefined, analytically rigorous CTF model can improve clinical decisions beyond established single-analyte assays and conventional assessment. Addressing this question will require prospective longitudinal studies, transparent integration methods, external validation, and demonstration of clinical utility at specific decision points.

## Figures and Tables

**Table 1:** Conceptual comparison of conventional liquid biopsy and the proposed CTF framework.

References	CTF Framework (Proposed)	Conventional Liquid Biopsy	Characteristic
[14,15,16]	Infer biologically active tumor and host states	Detect or quantify tumor-associated analytes	Main purpose
[14,15,16]	Multi-analyte, longitudinal, and multi-omic	Usually one analyte or molecular layer	Data structure
[14,15]	Interpretable functional scores (e.g., proliferation, immune evasion, metabolic stress, resistance)	Mutation, methylation, abundance, or cell count	Primary readout
[14]	Dynamic and response-oriented	Predominantly static or burden-oriented	Temporal interpretation
[14,15]	Network-level, mechanistically informed inference	Descriptive association	Biological interpretation
[14,16]	May improve through orthogonal signals, but requires prospective validation	Often limited by low tumor shedding	Early-disease sensitivity
[6,14]	Risk stratification, adaptive treatment, and early resistance detection	Detection, genotyping, and monitoring	Potential clinical role

Abbreviations: CTF, circulating tumor function.

**Table 2:** Representative molecular and multi-omic approaches in urogenital cancers, their potential clinical applications, and current level of translational evidence.

Cancer	Representative Molecular or Multi-Omic Approach	Potential Clinical Use	Level of Evidence/Translational Maturity	References
Prostate	Stemness-related multi-omic subtypes	Prognostic and treatment-response stratification	Exploratory/computational; retrospective cohort-based evidence	[70]
Prostate	DNA-damage-repair alterations and multi-omics profiling	PARP-inhibitor selection and resistance assessment	Mixed maturity: DDR genomic biomarkers are clinically implemented for PARP-inhibitor selection, whereas proteomic integration remains investigational	[72]
Bladder	GARS1-centered pan-cancer and multi-omic analysis	Prognostic hypothesis generation	Exploratory/computational; requires disease-specific prospective validation	[104]
Bladder	Sex-associated molecular and immune signatures	Sex-aware biomarker development and therapy stratification	Exploratory/retrospectively derived molecular signatures	[106]
Kidney	Programmed-cell-death patterns (anoikis and LDCD)	Prognostic and immunotherapy stratification; target discovery	Exploratory/computational; primarily retrospective cohort validation	[133]
Kidney	Proteogenomic c-MET activation in BAP1-deficient tumors	Context-specific therapeutic targeting	Translational/hypothesis-generating; molecular association demonstrated, but not clinically implemented as a patient-selection assay	[134]
Testicular	miR-371a-3p regulatory biomarker	Diagnosis and response monitoring	Advanced clinical validation; supported by prospective clinical evidence, but not yet universally implemented as standard-of-care	[146]

Abbreviations: BAP1, BRCA1-associated protein 1; DDR, DNA-damage repair; GARS1, glycyl-tRNA synthetase 1; LDCD, lysosome-dependent cell death; PARP, poly(ADP-ribose) polymerase.

## Data Availability

No new data were created or analyzed in this study. Data sharing is not applicable to this article.

## References

[1] Crosby D., Bhatia S., Brindle K.M., Coussens L.M., Dive C., Emberton M., Esener S., Fitzgerald R.C., Gambhir S.S., Kuhn P. (2022). Early detection of cancer. Science.

[2] Vaidyanathan R., Soon R.H., Zhang P., Jiang K., Lim C.T. (2018). Cancer diagnosis: From tumor to liquid biopsy and beyond. Lab Chip.

[3] Aredo J.V., Jamali A., Zhu J., Heater N., Wakelee H.A., Vaklavas C., Anagnostou V., Lu J. (2025). Liquid Biopsy Approaches for Cancer Characterization, Residual Disease Detection, and Therapy Monitoring. Am. Soc. Clin. Oncol. Educ. Book.

[4] Lone S.N., Nisar S., Masoodi T., Singh M., Rizwan A., Hashem S., El-Rifai W., Bedognetti D., Batra S.K., Haris M. (2022). Liquid biopsy: A step closer to transform diagnosis, prognosis and future of cancer treatments. Mol. Cancer.

[5] Pisapia P., Pepe F., Russo G., Capoluongo R., Coppola M., Giudice F.D., Ferro M., Madonna A., Musone M., Troncone G. (2025). Liquid biopsy testing in urological cancers: Focus on urine. Urol. Oncol..

[6] Youssef J., Yehya A., Salhab Z., Bitar R., Ghamlouche F., Bahmad H.F., Abou-Kheir W. (2025). Liquid biopsy in genitourinary cancers: Diagnostic and prognostic implications. World J. Clin. Oncol..

[7] Bergengren O., Pekala K.R., Matsoukas K., Fainberg J., Mungovan S.F., Bratt O., Bray F., Brawley O., Luckenbaugh A.N., Mucci L. (2023). 2022 Update on Prostate Cancer Epidemiology and Risk Factors-A Systematic Review. Eur. Urol..

[8] Wei J.T., Barocas D., Carlsson S., Coakley F., Eggener S., Etzioni R., Fine S.W., Han M., Kim S.K., Kirkby E. (2023). Early Detection of Prostate Cancer: AUA/SUO Guideline Part I: Prostate Cancer Screening. J. Urol..

[9] Campistol M., Lozano F., Carrion A., Raventós C.X., Morote J., Trilla E. (2025). Active Surveillance in Non-Muscle Invasive Bladder Cancer: A Systematic Review. Cancers.

[10] Makino T., Kadomoto S., Izumi K., Mizokami A. (2022). Epidemiology and Prevention of Renal Cell Carcinoma. Cancers.

[11] Nahouraii L.M., Allen J.L., Merrill S.B., Lehman E., Kaag M.G., Raman J.D. (2020). Histologic Heterogeneity of Extirpated Renal Cell Carcinoma Specimens: Implications for Renal Mass Biopsy. J. Kidney Cancer VHL.

[12] Yu S., Guo Z., Qiu Z., Wang L., Chen X., Xuan F. (2024). Global burden and trends of testicular cancer in adolescents and young adults from 1990 to 2021, with predictions to 2035. Sci. Rep..

[13] McHugh D.J., Gleeson J.P., Feldman D.R. (2024). Testicular cancer in 2023: Current status and recent progress. CA Cancer J. Clin..

[14] Ma L., Guo H., Zhao Y., Liu Z., Wang C., Bu J., Sun T., Wei J. (2024). Liquid biopsy in cancer current: Status, challenges and future prospects. Signal Transduct. Target. Ther..

[15] Abreu R.D.S., Ferreira D.D.P., de Araujo N.S., Horita S., Tilli T.M., Degrave W., Moreira A.D.S., Waghabi M.C. (2026). Liquid biopsy in cancer diagnosis and prognosis: A paradigm shift in precision oncology. Front. Mol. Biosci..

[16] Eskandar K. (2026). Liquid biopsy in genitourinary oncology: Current clinical applications and future prospects across prostate, bladder, and renal cancers. UroPrecision.

[17] Urbini M. (2026). Tumor Genomics and Liquid Biopsy in Cancer Biology: From Static Snapshots to Dynamic Measurements. Biomolecules.

[18] Li Y.Z., Kong S.N., Liu Y.P., Yang Y., Zhang H.M. (2023). Can Liquid Biopsy Based on ctDNA/cfDNA Replace Tissue Biopsy for the Precision Treatment of EGFR-Mutated NSCLC?. J. Clin. Med..

[19] Li L., Sun Y. (2024). Circulating tumor DNA methylation detection as biomarker and its application in tumor liquid biopsy: Advances and challenges. MedComm.

[20] Yu M., Stott S., Toner M., Maheswaran S., Haber D.A. (2011). Circulating tumor cells: Approaches to isolation and characterization. J. Cell Biol..

[21] Agashe R., Kurzrock R. (2020). Circulating Tumor Cells: From the Laboratory to the Cancer Clinic. Cancers.

[22] Saliminejad K., Khorram Khorshid H.R., Soleymani Fard S., Ghaffari S.H. (2019). An overview of microRNAs: Biology, functions, therapeutics, and analysis methods. J. Cell. Physiol..

[23] Boon R.A., Jaé N., Holdt L., Dimmeler S. (2016). Long Noncoding RNAs: From Clinical Genetics to Therapeutic Targets?. J. Am. Coll. Cardiol..

[24] Irmer B., Chandrabalan S., Maas L., Bleckmann A., Menck K. (2023). Extracellular Vesicles in Liquid Biopsies as Biomarkers for Solid Tumors. Cancers.

[25] Li B., Ming H., Qin S., Nice E.C., Dong J., Du Z., Huang C. (2025). Redox regulation: Mechanisms, biology and therapeutic targets in diseases. Signal Transduct. Target. Ther..

[26] Hong Y., Boiti A., Vallone D., Foulkes N.S. (2024). Reactive Oxygen Species Signaling and Oxidative Stress: Transcriptional Regulation and Evolution. Antioxidants.

[27] Park M.N., Kim M., Lee S., Kang S., Ahn C.H., Tallei T.E., Kim W., Kim B. (2025). Targeting Redox Signaling Through Exosomal MicroRNA: Insights into Tumor Microenvironment and Precision Oncology. Antioxidants.

[28] Song Y., Hou Z., Zhu L., Chen Y., Li J. (2025). Oxidative stress as a catalyst in prostate cancer progression: Unraveling molecular mechanisms and exploring therapeutic interventions. Discov. Oncol..

[29] Mas-Bargues C., Huete-Acevedo J., Arnal-Forné M., Sireno L., Pérez V., Borrás C. (2025). Extracellular Vesicles as Epigenetic Regulators of Redox Homeostasis: A Systematic Review and Meta-Analysis. Antioxidants.

[30] Zhao F., Xie H., Guan Y., Teng J., Li Z., Gao F., Luo X., Ma C., Ai X. (2024). A redox-related lncRNA signature in bladder cancer. Sci. Rep..

[31] Wang S., Shu J., Wang N., He Z. (2025). Exosomal non-coding RNAs: Mediators of crosstalk between cancer and cancer stem cells. Cell Death Discov..

[32] Hossam Abdelmonem B., Kamal L.T., Wardy L.W., Ragheb M., Hanna M.M., Elsharkawy M., Abdelnaser A. (2025). Non-coding RNAs: Emerging biomarkers and therapeutic targets in cancer and inflammatory diseases. Front. Oncol..

[33] AbdelHamid S.G., Halawa E.M., Ibrahim E.M., ElHefnawi M. (2026). Artificial intelligence-powered liquid biopsy in cancer: A paradigm shift in cancer detection and personalized care. Cancer Cell Int..

[34] Majka M. (2025). Non-coding RNAs as key player in cancer diagnosis and treatment. FEBS Open Bio.

[35] Belényesi S.K., Patmore S., O’Driscoll L. (2025). Extracellular vesicles and the tumour microenvironment. Biochim. Biophys. Acta Rev. Cancer.

[36] Zhao M., Huang W., Yang C., Lu L., Chen L., Wang J., Yang H., Guo Q., Qin T., Huang D. (2026). Research advances in extracellular vesicles for diagnosis and treatment of genitourinary cancers. Front. Cell Dev. Biol..

[37] Wang S., Wang Z., Liu M., Sun X. (2025). Role of extracellular vesicles in cancer: Implications in immunotherapeutic resistance. Front. Immunol..

[38] Aguilar-Cazares D., Perez-Medina M., Benito-Lopez J.J., Galicia-Velasco M., Meneses-Flores M., Camarena A., Lopez-Gonzalez J.S. (2026). The tumor microenvironment: Adding pieces to the puzzle. Front. Immunol..

[39] Pereira-Veiga T., Schneegans S., Pantel K., Wikman H. (2022). Circulating tumor cell-blood cell crosstalk: Biology and clinical relevance. Cell Rep..

[40] Yeat N.Y., Chen R.H. (2025). Extracellular vesicles: Biogenesis mechanism and impacts on tumor immune microenvironment. J. Biomed. Sci..

[41] Chen S., Zhang S., Shi Y., Liu X. (2026). Microenvironment crosstalk and immune evasion of circulating tumor cells: From mechanism to clinical significance. Chin. Med. J..

[42] Dou X., Feng C., Li J., Jiang E., Shang Z. (2025). Extracellular vesicle-mediated crosstalk in tumor microenvironment dominates tumor fate. Trends Cell Biol..

[43] Delrue C., Speeckaert M.M. (2026). Multi-Omics Integration in Urological Cancers: Unlocking Precision Diagnosis and Therapy Through Big Data. Arch. Esp. Urol..

[44] Eskandar K. (2026). Artificial intelligence and multi-omics integration in liquid biopsy for genitourinary cancers: A systematic scoping review. Int. Urol. Nephrol..

[45] Mao L., Wang H., Hu L.S., Tran N.L., Canoll P.D., Swanson K.R., Li J. (2025). Knowledge-Informed Machine Learning for Cancer Diagnosis and Prognosis: A Review. IEEE Trans. Autom. Sci. Eng..

[46] Olawade D.B., Oisakede E.O., Bello O.J., Analikwu C.C., Egbon E., Ojo A. (2026). Digital twins in oncology: From predictive modelling to personalised treatment strategies. Crit. Rev. Oncol. Hematol..

[47] Coman R.A., Nutu A., Strilciuc S., Budisan L., Berindan-Neagoe I. (2025). Molecular Cargo of Exosomes in Prostate Cancer: A Multi-Omics Perspective on Liquid Biopsies. Genes.

[48] Yaghoubi S.M., Zare E., Jafari Dargahlou S., Jafari M., Azimi M., Khoshnazar M., Shirjang S., Mansoori B. (2026). MicroRNAs in Prostate Cancer Liquid Biopsies: Early Detection, Prognosis, and Treatment Monitoring. Cells.

[49] Donovan M.J., Noerholm M., Bentink S., Belzer S., Skog J., O’Neill V., Cochran J.S., Brown G.A. (2015). A molecular signature of PCA3 and ERG exosomal RNA from non-DRE urine is predictive of initial prostate biopsy result. Prostate Cancer Prostatic Dis..

[50] McKiernan J., Donovan M.J., O’Neill V., Bentink S., Noerholm M., Belzer S., Skog J., Kattan M.W., Partin A., Andriole G. (2016). A Novel Urine Exosome Gene Expression Assay to Predict High-grade Prostate Cancer at Initial Biopsy. JAMA Oncol..

[51] McKiernan J., Donovan M.J., Margolis E., Partin A., Carter B., Brown G., Torkler P., Noerholm M., Skog J., Shore N. (2018). A Prospective Adaptive Utility Trial to Validate Performance of a Novel Urine Exosome Gene Expression Assay to Predict High-grade Prostate Cancer in Patients with Prostate-specific Antigen 2-10 ng/ml at Initial Biopsy. Eur. Urol..

[52] Tutrone R., Donovan M.J., Torkler P., Tadigotla V., McLain T., Noerholm M., Skog J., McKiernan J. (2020). Clinical utility of the exosome based ExoDx Prostate(IntelliScore) EPI test in men presenting for initial Biopsy with a PSA 2–10 ng/mL. Prostate Cancer Prostatic Dis..

[53] Rahbar K., Rosin R.D., Kidd M., Halim A.B., Sartor O. (2026). PROSTest, a Multigene Liquid Biopsy Signature, Effectively Stratifies Patients with High PSA for Prostate Biopsy. Prostate.

[54] Mazzetti S., Defeudis A., Nicoletti G., Chiorino G., De Luca S., Faletti R., Gatti M., Gontero P., Manfredi M., Mello-Grand M. (2024). Development and validation of a clinical decision support system based on PSA, microRNAs, and MRI for the detection of prostate cancer. Eur. Radiol..

[55] Chanhih N., Laraqui A., Hassine S., Ameur A., Hamedoun L., El Annaz H., Abi R., Tagajdid M.R., Amine I.L., Ennibi K. (2025). Circulating Tumor DNA as a Biomarker for Precision Medicine in Prostate Cancer: A Systematic Review. Int. J. Mol. Sci..

[56] Koo K.M., Phillips G., Srihari S., Farrell Á., Jaradi B., Fitzpatrick K.J., Yaxley J.W., Samaratunga H., Mainwaring P.N., Ru K.L. (2026). Rapid Decentralized Prostate Cancer Risk Stratification by Portable Liquid Biopsy Analysis within a Clinical Biosensor Validation Framework. Adv. Sci..

[57] Sighinolfi M.C., Pallotta G., Del Re M., Moosavi K., Schubert O., Rossi F., Gavi F., Assumma S., Panio E., Totaro A. (2026). Liquid Biopsy in Non-Metastatic Prostate Cancer: Clinical Evidence and Future Directions. Cancers.

[58] Mediavilla-Medel P., García-Simón N., González-Del-Alba A., Romero A. (2026). Liquid Biopsy in Advanced Prostate Cancer. Cancers.

[59] Goldkorn A., Tangen C., Plets M., Bsteh D., Xu T., Pinski J.K., Ingles S., Triche T.J., MacVicar G.R., Vaena D.A. (2024). Circulating Tumor Cell Count and Overall Survival in Patients with Metastatic Hormone-Sensitive Prostate Cancer. JAMA Netw. Open.

[60] Nørgaard M., Bjerre M.T., Fredsøe J., Vang S., Jensen J.B., De Laere B., Grönberg H., Borre M., Lindberg J., Sørensen K.D. (2023). Prognostic Value of Low-Pass Whole Genome Sequencing of Circulating Tumor DNA in Metastatic Castration-Resistant Prostate Cancer. Clin. Chem..

[61] Tukachinsky H., Madison R.W., Chung J.H., Gjoerup O.V., Severson E.A., Dennis L., Fendler B.J., Morley S., Zhong L., Graf R.P. (2021). Genomic Analysis of Circulating Tumor DNA in 3,334 Patients with Advanced Prostate Cancer Identifies Targetable BRCA Alterations and AR Resistance Mechanisms. Clin. Cancer Res..

[62] Casanova-Salas I., Athie A., Boutros P.C., Del Re M., Miyamoto D.T., Pienta K.J., Posadas E.M., Sowalsky A.G., Stenzl A., Wyatt A.W. (2021). Quantitative and Qualitative Analysis of Blood-based Liquid Biopsies to Inform Clinical Decision-making in Prostate Cancer. Eur. Urol..

[63] Armstrong A.J., Luo J., Nanus D.M., Giannakakou P., Szmulewitz R.Z., Danila D.C., Healy P., Anand M., Berry W.R., Zhang T. (2020). Prospective Multicenter Study of Circulating Tumor Cell AR-V7 and Taxane Versus Hormonal Treatment Outcomes in Metastatic Castration-Resistant Prostate Cancer. JCO Precis. Oncol..

[64] Fonseca N.M., Maurice-Dror C., Herberts C., Tu W., Fan W., Murtha A.J., Kollmannsberger C., Kwan E.M., Parekh K., Schönlau E. (2024). Prediction of plasma ctDNA fraction and prognostic implications of liquid biopsy in advanced prostate cancer. Nat. Commun..

[65] He J.X., Li L., Chen S., Chen R.J., Zhuang J.L., Liu C., Burrow W., Chang J., Tanziela T., Bonfil R.D. (2026). Outsmarting Metastatic Prostate Cancer: Integration of Imaging, Liquid Biopsies and Biomarkers with Artificial Intelligence. Technol. Cancer Res. Treat..

[66] Kim H., Park K.U. (2023). Clinical Circulating Tumor DNA Testing for Precision Oncology. Cancer Res. Treat..

[67] de Bono J., Mateo J., Fizazi K., Saad F., Shore N., Sandhu S., Chi K.N., Sartor O., Agarwal N., Olmos D. (2020). Olaparib for Metastatic Castration-Resistant Prostate Cancer. N. Engl. J. Med..

[68] Annala M., Vandekerkhove G., Khalaf D., Taavitsainen S., Beja K., Warner E.W., Sunderland K., Kollmannsberger C., Eigl B.J., Finch D. (2018). Circulating Tumor DNA Genomics Correlate with Resistance to Abiraterone and Enzalutamide in Prostate Cancer. Cancer Discov..

[69] Kalinina E.V., Gavriliuk L.A., Pokrovsky V.S. (2022). Oxidative Stress and Redox-Dependent Signaling in Prostate Cancer. Biochemistry.

[70] Zheng K., Hai Y., Xi Y., Zhang Y., Liu Z., Chen W., Hu X., Zou X., Hao J. (2023). Integrative multi-omics analysis unveils stemness-associated molecular subtypes in prostate cancer and pan-cancer: Prognostic and therapeutic significance. J. Transl. Med..

[71] Li S., Tian Y., Sun Y., Xu T., Wang W. (2025). Molecular stratification of prostate cancer through sensory perception-related multi-omics analysis reveals chemoresistant mechanisms. Cell. Oncol..

[72] Fatima Y., Jobre K.N., Gomez-Gomez E., Małkiewicz B., Vlahou A., Mokou M., Mischak H., Frantzi M., Jankowski V. (2025). Omics-Mediated Treatment for Advanced Prostate Cancer: Moving Towards Precision Oncology. Int. J. Mol. Sci..

[73] Trenti E., D’Elia C., Mian C., Schwienbacher C., Hanspeter E., Pycha A., Kafka M., Degener S., Danuser H., Roth S. (2019). Diagnostic predictive value of the Bladder EpiCheck test in the follow-up of patients with non-muscle-invasive bladder cancer. Cancer Cytopathol..

[74] D’Andrea D., Soria F., Zehetmayer S., Gust K.M., Korn S., Witjes J.A., Shariat S.F. (2019). Diagnostic accuracy, clinical utility and influence on decision-making of a methylation urine biomarker test in the surveillance of non-muscle-invasive bladder cancer. BJU Int..

[75] Pichler R., Fritz J., Tulchiner G., Klinglmair G., Soleiman A., Horninger W., Klocker H., Heidegger I. (2018). Increased accuracy of a novel mRNA-based urine test for bladder cancer surveillance. BJU Int..

[76] Kavcic N., Peric I., Zagorac A., Kokalj Vokac N. (2022). Clinical Evaluation of Two Non-Invasive Genetic Tests for Detection and Monitoring of Urothelial Carcinoma: Validation of UroVysion and Xpert Bladder Cancer Detection Test. Front. Genet..

[77] Pepe L., Fiorentino V., Pizzimenti C., Riganati G., Franchina M., Micali M., Russotto F., Ieni A., Tuccari G., Fadda G. (2024). The Simultaneous Use of Bladder Epicheck^®^ and Urinary Cytology Can Improve the Sensitivity and Specificity of Diagnostic Follow-Up of Urothelial Lesions: Up-to-Date Data from a Multi-Institutional Cohort. Diseases.

[78] D’Elia C., Folchini D.M., Mian C., Hanspeter E., Schwienbacher C., Spedicato G.A., Pycha S., Vjaters E., Degener S., Kafka M. (2021). Diagnostic value of Xpert^®^ Bladder Cancer Monitor in the follow-up of patients affected by non-muscle invasive bladder cancer: An update. Ther. Adv. Urol..

[79] Lotan Y., Daneshmand S., Shore N., Black P., Scarpato K.R., Patel A., Lough T., Shoskes D.A., Raman J.D. (2024). A Multicenter Prospective Randomized Controlled Trial Comparing Cxbladder Triage to Cystoscopy in Patients with Microhematuria: The Safe Testing of Risk for Asymptomatic Microhematuria Trial. J. Urol..

[80] Raman J.D., Kavalieris L., Konety B., Porten S., Daneshmand S., Lotan Y., Loo R. (2021). The Diagnostic Performance of Cxbladder Resolve, Alone and in Combination with Other Cxbladder Tests, in the Identification and Priority Evaluation of Patients at Risk for Urothelial Carcinoma. J. Urol..

[81] Ward D.G., Baxter L., Ott S., Gordon N.S., Wang J., Patel P., Piechocki K., Silcock L., Sale C., Zeegers M.P. (2023). Highly Sensitive and Specific Detection of Bladder Cancer via Targeted Ultra-deep Sequencing of Urinary, DNA. Eur. Urol. Oncol..

[82] Batista R., Vinagre J., Prazeres H., Sampaio C., Peralta P., Conceição P., Sismeiro A., Leão R., Gomes A., Furriel F. (2019). Validation of a Novel, Sensitive, and Specific Urine-Based Test for Recurrence Surveillance of Patients with Non-Muscle-Invasive Bladder Cancer in a Comprehensive Multicenter Study. Front. Genet..

[83] Chen X., Zhang J., Ruan W., Huang M., Wang C., Wang H., Jiang Z., Wang S., Liu Z., Liu C. (2020). Urine DNA methylation assay enables early detection and recurrence monitoring for bladder cancer. J. Clin. Investig..

[84] Wu X., Zhou Y., Chen J., Zhang H., Zhang Q., Hao T., Guo Z. (2026). A high-throughput photothermal aptasensing platform using MnO_2_@CuS nanoprobes for noninvasive detection of bladder cancer cells in simulated liquid biopsy. Mikrochim. Acta.

[85] Dayati P., Shakhssalim N., Allameh A. (2024). Over-expression of KRT17 and MDK genes at mRNA levels in urine-exfoliated cells is associated with early non-invasive diagnosis of non-muscle-invasive bladder cancer. Clin. Biochem..

[86] Qi W., Qisheng L., Weiyang W., Yuan L., Linfeng L., Bin C., Yaqiang H. (2025). Integrating urinary dual-gene methylation and VI-RADS score to predict residual tumors after TURBT in NMIBC: A nomogram-based model. BMC Urol..

[87] Murakami T., Minami K., Harabayashi T., Maruyama S., Takada N., Kashiwagi A., Miyata H., Sato Y., Matsumoto R., Kikuchi H. (2024). Cross-sectional and longitudinal analyses of urinary extracellular vesicle mRNA markers in urothelial blad-der cancer patients. Sci. Rep..

[88] Zhao L., Li J., Xue Z., Wang J. (2024). Exosomal noncoding RNAs asnoninvasive biomarkers in bladder cancer: A diagnostic meta-analysis. Clin. Transl. Oncol..

[89] Long C., Shi H., Li J., Chen L., Lv M., Tai W., Wang H., Xu Y. (2024). The diagnostic accuracy of urine-derived exosomes for bladder cancer: A sys-tematic review and meta-analysis. World J. Surg. Oncol..

[90] Greenberg Z.F., Hutchinson T., Kahn J., Graim K.S., O’Malley P., He M. (2026). Urinary Extracellular Vesicles for High-Precision Bladder Cancer Subtyping and Prognosis. J. Extracell. Biol..

[91] Shi W.Y., Liu K.J., Esfahani M.S., Mach K.E., Phillips N.A., Almanza D., Bajpai R.K., Schroers-Martin J.G., Trabanino L., Lee T.J. (2026). Field-effect-informed urine liquid biopsy for bladder cancer. Cell.

[92] Christensen E., Nordentoft I., Birkenkamp-Demtröder K., Elbæk S.K., Lindskrog S.V., Taber A., Andreasen T.G., Strandgaard T., Knudsen M., Lamy P. (2023). Cell-Free Urine and Plasma DNA Mutational Analysis Predicts Neoadjuvant Chemotherapy Response and Outcome in Patients with Muscle-Invasive Bladder Cancer. Clin. Cancer Res..

[93] Xue Z., Qie Y., Shen C., Wu Z., Chen H., Lin Y., Li R., Huang S., Hu H. (2026). Urinary Tumor DNA to Identify Candidates for Repeat Transurethral Resection in Non-Muscle-Invasive Bladder Cancer. Cancer Sci..

[94] Powles T., Chang Y.H., Yamamoto Y., Munoz J., Reyes-Cosmelli F., Peer A., Cohen G., Yu E.Y., Lorch A., Bavle A. (2024). Pembrolizumab for advanced urothelial carcinoma: Exploratory ctDNA biomarker analyses of the KEYNOTE-361 phase 3 trial. Nat. Med..

[95] Powles T., Assaf Z.J., Degaonkar V., Grivas P., Hussain M., Oudard S., Gschwend J.E., Albers P., Castellano D., Nishiyama H. (2024). Updated Overall Survival by Circulating Tumor DNA Status from the Phase 3 IMvigor010 Trial: Adjuvant Atezolizumab Versus Observation in Muscle-invasive Urothelial Carcinoma. Eur. Urol..

[96] Stewart T.F., Chalfin H., Simon N., Tan A., Apolo A., McKay R.R. (2024). Perioperative Use of ctDNA to Guide Treatment for Urothelial Carcinoma: The Future is Now. Bladder Cancer.

[97] Dyrskjøt L., Birkenkamp-Demtröder K., Nordentoft I., Strandgaard T., Lindskrog S.V., Milling R.V., Körner S.K., Brandt S.B., Knudsen M., Andreasen T.G. (2026). ctDNA-guided immunotherapy following radical cystectomy for muscle-invasive bladder cancer: Results from the TOMBOLA trial. Ann. Oncol..

[98] Gulati G.S., Vasseur D., Nawfal R., Sotudian S., Semaan K., Eid M., Seo J.H., Phillips N., Canniff J.J., Savignano H. (2026). Integrated inference of cancer gene expression from cell-free plasma chromatin. bioRxiv.

[99] Duquesne I., Epelbaum I., Prost D., Haberstich M., Suartz C.V., Blons H., Taly V., Laurent-Puig P., Horowitz A., Sfakianos J.P. (2026). Blood and Urine Circulating Tumor DNA in Urothelial Bladder Cancer: State of the Art and Clinical Perspective. Eur. Urol. Oncol..

[100] Zeng D., Liu B., Deng F., Wang Y., Liu J., Deng Z. (2026). Multidimensional liquid biopsy in bladder cancer: Advances in circulating tumor cells, circulating tumor DNA, exosomes, and metabolomics. Oncologist.

[101] Jelic M.D., Mandic A.D., Maricic S.M., Srdjenovic B.U. (2021). Oxidative stress and its role in cancer. J. Cancer Res. Ther..

[102] Dugbartey G.J., Relouw S., McFarlane L., Sener A. (2024). Redox System and Oxidative Stress-Targeted Therapeutic Approaches in Bladder Cancer. Antioxidants.

[103] Kamoun A., de Reyniès A., Allory Y., Sjödahl G., Robertson A.G., Seiler R., Hoadley K.A., Groeneveld C.S., Al-Ahmadie H., Choi W. (2020). A Consensus Molecular Classification of Muscle-invasive Bladder Cancer. Eur. Urol..

[104] Liu W., Wei C., He Q., Chen Z., Zhuang W., Guo Y., Xue X. (2024). Multiple omics integrative analysis identifies GARS1 as a novel prognostic and immunological biomarker: From pan-cancer to bladder cancer. Sci. Rep..

[105] Xu Y., Sun X., Liu G., Li H., Yu M., Zhu Y. (2024). Integration of multi-omics and clinical treatment data reveals bladder cancer therapeutic vulnerability gene combinations and prognostic risks. Front. Immunol..

[106] Wang Y., Bhandary P., Griffin K., Moore J.H., Li X., Wang Z.P. (2025). Integrative multi-omics study identifies sex-specific molecular signatures and immune modulation in bladder cancer. Front. Bioinform..

[107] Rajandram R., Suren Raj T.L., Gobe G.C., Kuppusamy S. (2025). Liquid biopsy for renal cell carcinoma. Clin. Chim. Acta.

[108] Orsatti A., Fernandes-Pontes F., ESilva J.R., Tavares N.T., Jerónimo C., Henrique R., Rodrigues Â., Ricci C., Lobo J. (2026). Advances in Renal Cell Carcinoma Diagnosis: A Review on Biomarkers. Pathobiology.

[109] Netti G.S., De Luca F., Camporeale V., Khalid J., Leccese G., Troise D., Sanguedolce F., Stallone G., Ranieri E. (2025). Liquid Biopsy as a New Tool for Diagnosis and Monitoring in Renal Cell Carcinoma. Cancers.

[110] Yip W., Hakimi A.A. (2023). Circulating Tumor DNA (ctDNA) in Kidney Cancer: A Narrative Review. Soc. Int. Urol. J..

[111] Patel K.R., Rais-Bahrami S., Basu A. (2024). High sensitivity ctDNA assays in genitourinary malignancies: Current evidence and future directions. Oncologist.

[112] Ben-David R., Alerasool P., Kalola H., Tillu N., Almoflihi M., Tsao C.K., Galsky M.D., Sfakianos J.P., Wiklund P., Waingankar N. (2024). Tumor Characteristics Associated with Preoperatively Detectable Tumor-Informed Circulating Tumor DNA in Patients with Renal Masses Suspicious for Renal Cell Carcinoma. JCO Precis. Oncol..

[113] Ito K., Braun D.A. (2026). Next-generation liquid biopsies: Detecting circulating epigenetic changes to identify translocation renal cell carcinoma. J. Clin. Investig..

[114] Garinet S., Semaan K., Li J., Zhang Z., Konda P., Sadagopan A., Canniff J., Phillips N., Klega K., Pandey M. (2026). Cell-free DNA epigenomic profiling enables noninvasive detection and monitoring of translocation renal cell carcinoma. J. Clin. Investig..

[115] Le L.N., Munir J., Kim E.B., Ryu S. (2024). Kidney Cancer and Potential Use of Urinary Extracellular Vesicles. Oncol. Rev..

[116] Li N., Zhang H., Hu K., Chu J. (2021). A novel long non-coding RNA-based prognostic signature for renal cell carcinoma patients with stage IV and histological grade G4. Bioengineered.

[117] Vannuccini G., Paladini A., Mearini M., Cocci F., Giardino G., Mangione P., Maulà V., Mirra D., Mearini E., Cochetti G. (2026). Liquid Biopsy in Clear Cell Renal Cell Carcinoma: Diagnostic Potential of Urinary miRNAs. Cancers.

[118] Marigliano C., Badia S., Bellini D., Rengo M., Caruso D., Tito C., Miglietta S., Palleschi G., Pastore A.L., Carbone A. (2019). Radiogenomics in Clear Cell Renal Cell Carcinoma: Correlations Between Advanced CT Imaging (Texture Analysis) and MicroRNAs Expression. Technol. Cancer Res. Treat..

[119] Mytsyk Y., Kowal P., Kobilnyk Y., Lesny M., Skrzypczyk M., Stroj D., Dosenko V., Kucheruk O. (2025). Machine learning-assisted radiogenomic analysis for miR-15a expression prediction in renal cell carcinoma. BMC Cancer.

[120] Peng Y.L., Yu B., Huang T.X., Zhou Z.H., Zhang H., Tang W.X.F., Xu X.X., Zhu D.Q., Yang R.W., Bao H. (2025). Early detection of renal cell carcinoma: A novel cell-free DNA fragmentomics-based liquid biopsy assay. ESMO Open.

[121] Liang S.I., Quandt Z., Wienke S., Wang J., Gordon S., Barnett R.M., Masannat J., Chang K., Espenschied C.R., Quinn K.J. (2025). Methylation-Based ctDNA Tumor Fraction Changes Predict Long-Term Clinical Benefit from Immune Checkpoint Inhibitors in RADIOHEAD, a Real-World Pan-Cancer Study. Cancer Res. Commun..

[122] Basu A., Au C., Kommalapati A., Kandala H., Sudhaman S., Mahmood T., Carson C., Pajak N., Dutta P., Calhoun M. (2024). Longitudinal Testing of Circulating Tumor DNA in Patients with Metastatic Renal Cell Carcinoma. JCO Precis. Oncol..

[123] Chehrazi-Raffle A., Muddasani R., Dizman N., Hsu J., Meza L., Zengin Z.B., Malhotra J., Chawla N., Dorff T., Contente-Cuomo T. (2023). Ultrasensitive Circulating Tumor DNA Pilot Study Distinguishes Complete Response and Partial Response with Immunotherapy in Patients with Metastatic Renal Cell Carcinoma. JCO Precis. Oncol..

[124] Lee S.A., Choi C., Yoo T.H. (2021). Extracellular vesicles in kidneys and their clinical potential in renal diseases. Kidney Res. Clin. Pract..

[125] Li Q., Tian J., Chen C., Liu H., Li B. (2024). Meta-analysis of the diagnostic value of exosomal microRNAs in renal cell carcinoma. Front. Oncol..

[126] Omri L., Naigeon M., Flippot R., Gavira-Díaz J., Poveda-Ferriols J., Nguyen D., Abdi C., Arroyo-Salgado A., Chaput N., de Velasco G. (2024). Blood-based circulating biomarkers for prediction of immune-checkpoint inhibitors efficacy in renal cell carcinoma. Explor. Target. Antitumor Ther..

[127] Mack P.C., Miao J., Redman M.W., Moon J., Goldberg S.B., Herbst R.S., Melnick M.A., Walther Z., Hirsch F.R., Politi K. (2022). Circulating Tumor DNA Kinetics Predict Progression-Free and Overall Survival in EGFR TKI-Treated Patients with EGFR-Mutant NSCLC (SWOG S1403). Clin. Cancer Res..

[128] Correa A.F., Kalashnikova E., Wu H.T., Winters R.M., Balcioglu M., Sudhaman S., Connolly D.C., Gong Y., Uzzo R.G., Sethi H. (2024). Association of circulating tumor DNA with patient prognosis in surgically resected renal cell carcinoma. Oncologist.

[129] Coca Membribes S., Xu W., Suárez C., Patel P.M., Larkin J., Valderrama B.P., Mein C., Ackermann C., Powles T. (2026). KIM-1 in Advanced Papillary and Clear Cell Renal Cell Carcinoma. Eur. Urol..

[130] Jin Z., Hu B., Zhu S., Fu S., Deng Z., Wang C., Liu J., Wang T., Wu Y. (2025). Exploration of oxidative stress-related molecular signature for clear cell renal cell carcinoma. Discov. Oncol..

[131] Zhang Y., Zhang S., Sun H., Xu L. (2025). The pathogenesis and therapeutic implications of metabolic reprogramming in renal cell carcinoma. Cell Death Discov..

[132] Jomova K., Alomar S.Y., Valko R., Fresser L., Nepovimova E., Kuca K., Valko M. (2026). Interplay of oxidative stress and antioxidant mechanisms in cancer development and progression. Arch. Toxicol..

[133] Ou F., Pan Y., Chen Q., Zeng L., Wei K., Liu D., Guo Q., Zhou L., Yang J. (2025). Integrating machine learning and multi-omics analysis to unveil key programmed cell death patterns and immunotherapy targets in kidney renal clear cell carcinoma. Sci. Rep..

[134] Du B., Zhou Y., Li W., He H., Chen M., Feng N. (2024). Proteogenomics identifies c-Met inhibition as a therapeutic strategy for BAP1-deficient clear cell renal cell carcinoma. Mol. Biomed..

[135] Gerke M.B., Jansen C.S., Bilen M.A. (2024). Circulating Tumor DNA in Genitourinary Cancers: Detection, Prognostics, and Therapeutic Implications. Cancers.

[136] Krasic J., Skara L., Bojanac A.K., Ulamec M., Jezek D., Kulis T., Sincic N. (2022). The utility of cfDNA in TGCT patient management: A systematic review. Ther. Adv. Med. Oncol..

[137] Sykes J., Kaldany A., Jang T.L. (2024). Current and Evolving Biomarkers in the Diagnosis and Management of Testicular Germ Cell Tumors. J. Clin. Med..

[138] Esposito F., De Martino M., Franco V., Fusco A., Chieffi P. (2025). Potential therapeutic targets and biomarkers in testicular germ cell tumor oncogenesis. Expert. Opin. Ther. Targets.

[139] Nastały P., Honecker F., Pantel K., Riethdorf S. (2021). Detection of Circulating Tumor Cells (CTCs) in Patients with Testicular Germ Cell Tumors. Methods Mol. Biol..

[140] Nastały P., Ruf C., Becker P., Bednarz-Knoll N., Stoupiec M., Kavsur R., Isbarn H., Matthies C., Wagner W., Höppner D. (2014). Circulating tumor cells in patients with testicular germ cell tumors. Clin. Cancer Res..

[141] Oliveira-Lopes B., Tavares N.T., Lobo J. (2026). Testicular germ cell tumors and molecular biomarkers. Curr. Opin. Urol..

[142] Fern L.A., Greenwood M., Smith S., Brand S., Coleman N., Stark D.P., Murray M.J. (2021). Pre-Implementation Assessment of the Acceptability of Using Circulating microRNAs for Follow-Up of Malignant Germ-Cell Tumors. Clin. Genitourin. Cancer.

[143] Murray M.J., Huddart R.A., Coleman N. (2016). The present and future of serum diagnostic tests for testicular germ cell tumours. Nat. Rev. Urol..

[144] Lobo J., Acosta A.M., Netto G.J. (2023). Molecular Biomarkers with Potential Clinical Application in Testicular Cancer. Mod. Pathol..

[145] Bumbasirevic U., Bojanic N., Simic T., Milojevic B., Zivkovic M., Kosanovic T., Kajmakovic B., Janicic A., Durutovic O., Radovanovic M. (2022). Interplay between Comprehensive Inflammation Indices and Redox Biomarkers in Testicular Germ-Cell Tumors. J. Pers. Med..

[146] Dieckmann K.P., Radtke A., Geczi L., Matthies C., Anheuser P., Eckardt U., Sommer J., Zengerling F., Trenti E., Pichler R. (2019). Serum Levels of MicroRNA-371a-3p (M371 Test) as a New Biomarker of Testicular Germ Cell Tumors: Results of a Prospective Multicentric Study. J. Clin. Oncol..

[147] Praharaj P.P., Bhutia S.K., Nagrath S., Bitting R.L., Deep G. (2018). Circulating tumor cell-derived organoids: Current challenges and promises in medical research and precision medicine. Biochim. Biophys. Acta Rev. Cancer.

[148] Dang D.K., Park B.H. (2022). Circulating tumor DNA: Current challenges for clinical utility. J. Clin. Investig..

[149] Coppola C.A., De Summa S., Matera G., Pilato B., Traversa D., Tommasi S. (2025). Liquid Biopsy: The Challenges of a Revolutionary Approach in Oncology. Int. J. Mol. Sci..

